# Do magnification loupes affect the precision of cavity preparations made by undergraduates? A randomized crossover study

**DOI:** 10.1186/s12903-022-02232-z

**Published:** 2022-05-19

**Authors:** Florin Eggmann, Delia R. Irani, Patrizia A. Fehlbaum, Klaus W. Neuhaus

**Affiliations:** 1 Department of Periodontology, Endodontology and Cariology, University Center for Dental Medicine UZB, University of Basel, Mattenstrasse 40, 4058 Basel, Switzerland; 2 Department of Preventive, Restorative and Pediatric Dentistry, University of Bern, Bern, Switzerland

**Keywords:** Visual acuity, Magnification device, Dental education, Undergraduate training, Restorative dentistry

## Abstract

**Background:**

Evidence on the effect of magnification devices on procedure quality in restorative dentistry is scant. This study therefore aimed to assess, under simulated clinical conditions, if magnification loupes affect the quality of preparations carried out by undergraduate dental students.

**Methods:**

59 undergraduate dental students underwent two visual acuity tests, based on which they were divided into a “low visual acuity group” (visus < 1) and a “good visual acuity group” (visus ≥ 1). In a randomized crossover experiment, participants performed a two-dimensional S and a three-dimensional O figure preparation with a dental handpiece on standardized acrylic blocs designed for preclinical restorative training. Each participant carried out the preparation tasks twice, once with magnification loupes (2.5×) and once without. Two blinded investigators independently evaluated parameters of preparation precision. Data were analyzed using Spearman rank correlation coefficients, intra-class correlation coefficients, and Wilcoxon rank-sum tests (α = 0.05).

**Results:**

Participants from the “low visual acuity group” did not show a statistically significant improvement in accuracy when they used magnification loupes for the S figure preparation (*p* ≥ 0.0625). Participants from the “high visual acuity group” obtained a higher level of accuracy (*p* ≤ 0.0012) when they used magnification loupes for the S figure preparation. The use of magnification loupes had no statistically significant effect on the accuracy parameters of the O figure cavity preparations (*p* ≥ 0.1865). Participants with high visual acuity achieved only a marginally better accuracy than participants with a visus < 1.

**Conclusions:**

This study suggests that loupes with 2.5× magnification increase the accuracy of two-dimensional preparations while they have no significant effect, favorable or otherwise, on the accuracy of complex, three-dimensional cavity preparations of untrained dental students.

## Background

The last decades have seen a notable increase in the number of dental professionals who use magnification loupes [1, 2]. Magnification devices offer some ergonomic benefits to dental practitioners [2]. In addition, magnification can reliably compensate for presbyopic decreases in visual acuity (defined as the ability to recognize small details with precision), which usually set in around the age of 40 years [3].

Magnification devices are of particular benefit for endodontic procedures [4–6]. For instance, using an operating microscope or magnification loupes is essential to locate the orifice of the second mesiobuccal canal of maxillary molars [7]. In restorative dentistry, by contrast, the merits of assisted vision are less clear. Though some expert opinion pieces and case reports suggest that the use of magnification devices enhances the quality of restorative procedures [8, 9], higher level evidence is currently scanty and inconclusive. While some studies, simulating clinical conditions, indicated that magnification loupes improve the quality of restorative work [10–12], other investigations reported no such improvement [13–16].

The primary objective of this study was therefore to assess, under simulated clinical conditions, if magnification loupes affect the quality of preparations carried out by undergraduate dental students. The secondary objective of this study was to evaluate whether two different visual acuity tests show a correlation with the participants’ preparation accuracy. The null hypotheses were that magnification loupes would not affect the quality of preparation made by undergraduates and that the undergraduates’ visual acuity would not correlate with their preparation accuracy.

## Methods

### Ethical approval and informed consent

The local ethics review board, Cantonal Ethics Committee Bern, Switzerland, granted the study exemption from oversight since neither patients nor patients' data were involved. The study was carried out in accordance the regulatory requirements of the Swiss Human Research Act and Human Research Ordinance. Informed consent was obtained from all undergraduate dental students, who participated voluntarily in the study. They agreed to the use of generated data for research and educational purposes. The study, conducted without compulsory attendance as part of the undergraduates’ regular pre-clinical course in restorative dentistry, was not registered in a publicly accessible primary register that participates in the WHO International Clinical Trial Registry Platform because the investigation did not involve any biomedical or behavioral intervention or treatment.

### Participants

Two consecutive undergraduate classes, comprising 59 undergraduate dental students in total, voluntarily took part in this study. Table 1 reports the age distribution of the participants. The participants had two months of preclinical restorative training in the dental skill lab as part of their regular curriculum prior to the study. These two months of preclinical restorative training comprised a total of 16 four-hour, supervised sessions dedicated to adhesive cavity preparations and direct resin-based composite restorations (class I, two sessions; class II, seven sessions; class III and IV, five sessions; class V, two sessions). The undergraduates were not familiar with working with dental magnification loupes.

**Table 1:** Age distribution of undergraduate dental students participating in the study

Age (years)	N
20–25	47
26–30	8
31–35	2
36–40	0
41–45	0
46–50	2

### Visual acuity measurements

To measure the participants’ visual acuity, two tests were employed. The tests were performed by a board-certified optician under standardized viewing conditions. Participants with corrective eyeglasses or contact lenses took the visual acuity tests with their corrective lenses. The binocular visual acuity of the participants was recorded using the Snellen chart at a distance of 5 m. Each eye was tested independently. In addition, the participants took the near visual acuity test according to Eichenberger et al. [3] A detailed description of this test has been reported previously [3]. In brief, the visual acuity was measured with a miniaturized visual test chart, featuring E optotypes 0.05–0.5 mm in size, at a fixed distance of 300 mm. For each test separately, the participants were divided into a “low visual acuity group,” defined by a visus < 1, and a “good visual acuity group”, defined by a visus ≥ 1. This threshold was selected based on previous studies assessing the visual acuity of dental practitioners [3, 17].

### Preparation procedure

**Fig. 1:**
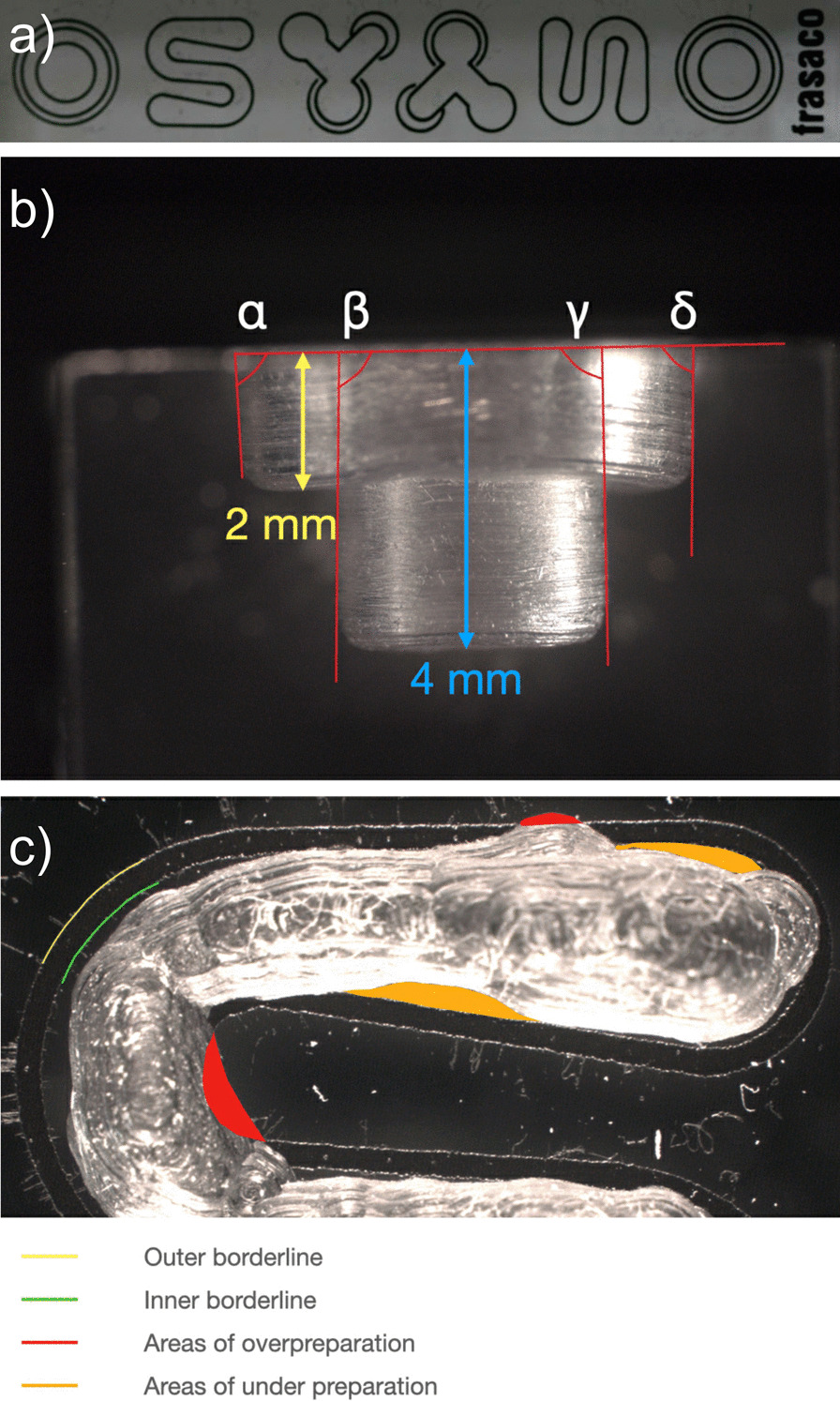
**a** Standardized acrylic bloc designed for preclinical restorative training. Only the O and S figures were assessed in the present study. **b** Preparation precision assessment based on side view photographs of the O figure: the evenness and the accuracy of depth of the cavities were evaluated by taking length measurements. The internal angles (α, β, γ, and δ) of the axial walls of the preparation were measured to assess the compliance with the required angles. **c** Preparation precision assessment based on top view photographs of the S figure: the parameter overpreparation and the parameter underpreparation quantified the area (red) outside the borderline and the area (yellow) inside the borderline of the S figure, respectively

Each participant received two standardized, mass-produced, clear acrylic blocs (A-PTM 99 001, Frasaco GmbH, Tettnang, Germany). These acrylic blocs were designed for preclinical restorative training and featured three geometric figures, each in duplicate (an O figure, a S figure, and a Y figure) (Fig. 1a). The participants had a water-cooled, high-speed contra-angle handpiece (KaVo EXPERTmatic E25 L, KaVo Dental AG, Kloten, Switzerland) with a cylindrical diamond at their disposal. Constant water-cooling was compulsory for the work with the handpiece. The diamond bur had a shank length of 6 mm, a rounded edge, a diameter of the working part of 1.3 mm, and a grit size of 80 µm (ISO 314 157 524 013, 836KR, Intensiv SA, Montagnola, Switzerland). The participants were given an instruction, supported by a Microsoft PowerPoint presentation, on the two tasks they should fulfill immediately afterwards. The first task required the participants to remove the acrylic within the black borderline of the S figure as accurately and completely as possible without crossing the borderline. The depth of the preparation did not matter, i.e., the preparation precision assessment of the S figure took account of two-dimensional parameters. The second task required the participants to remove the acrylic of the O figure: its outer circle to a depth of 2 mm and its inner circle to a depth of 4 mm. The cavity walls were expected to be perpendicular to the surface of the block and the cavity floors as even as possible. To control the depth of the preparation, the participants had transparent 1 mm scaled foil available at their workstation. Whether or not the black borderlines were crossed was inconsequential in the second task. In the O figure preparation, parameters of the three-dimensional extent of the cavity were assessed regarding their compliance with the preparation guideline.

Each participant had to perform the tasks twice, once with magnification loupes and once without. Based on a randomized allocation scheme, generated with online freeware (www.randomizer.org), one half of the participants started with the magnification loupes first while the other half started without the magnification loupes. Galilean magnification loupes with a magnification level of 2.5× and a working distance of 300 mm (EyeMag, Carl Zeiss AG, Jena, Germany) were custom fitted for each participant to ensure an optimal fit and customization according to the pupillary distance. Participants with corrective lenses wore the same eyeglasses or contact lenses as they did when they took the visual acuity tests. Participants without corrective eyeglasses wore safety spectacles. The loupes were fixed on headbands, which allowed the participants to wear their corrective eyeglasses or safety spectacles while working with the loupes.

The experiments took place at the work benches in the dental skills lab. Each workstation had a dental operating light. To ensure a constant working distance of 300 mm throughout the experiments, the eyewear, including eyeglasses, safety spectacles, and the magnification loupes, was tied with a piece of non-elastic string to the fixed support tubes of the dental operating lights above the participants’ heads. Supervisors, one per four participants, oversaw the experiments, monitoring that the participants always kept the working distance.

The experiments with and without the magnification loupes were carried out in a row, with only a short break in between to put in place the new sets of eyewear (magnification loups for one half of the participants, corrective eyeglasses, or safety spectacles for the other). Each experiment, the one with and the one without the magnification loupes, lasted 60 min. During the first 15 min of each experiment, the participants could practice on the duplicate S and O figures, which would not be assessed. In the remaining 45 min, the participants performed the preparations, which would later be assessed. Participants who wished to hand in their preparations ahead of time were free to do so.

### Preparation accuracy assessments

A standardized top view photograph of each acrylic bloc was taken with a light microscope (Leica M 430, Leica, Heerbrugg, Switzerland) equipped with a camera (Leica DFC 495, Leica) at a magnification level of 2.5×. Additionally, two perpendicular side view photographs, orthogonal to one another, of each acrylic bloc were made at the same magnification level. The TIFF files of the photographs were imported to an image processing program (ImageJ [1.51v 9], National Institutes of Health, Bethesda, MD, USA), which was used for the quantitative accuracy assessments. Two investigators independently carried out the measurements in a blinded way.

The assessment of each O figure preparation was performed on the two corresponding side view photographs. Four measurement parameters were recorded: the depth of the cavities, the evenness of the cavity floors, presence of any ledges along the axial walls, and the internal angles of the axial walls of the preparation.

Twenty-four length measurements, 12 per side view photograph, were obtained of each preparation. Half of the length measurements recorded the distance from the top surface to the level of the outer cavity floor, the other half recorded the distance from the top surface to the level of the inner cavity floor (Fig. 1b). The length measurements were spaced out as evenly as feasible across the entire width of the preparation.

An estimate of the evenness of each cavity floor was made by calculating the difference between the corresponding highest and the lowest length measurements (the best possible value was 0 mm) and by calculating the arithmetic mean of the 12 corresponding length measurements (the best possible values were 2 mm and 4 mm for the outer and the inner circle, respectively).

The presence of any ledges along the axial walls was assessed semiquanitatively by recording the number of conspicuous ledges for each preparation.

The internal angles of the axial walls of the preparation were measured on each photograph (Fig. 1b). The difference of each angle measure and 90° (the ideal angular dimension) was calculated and subsequently the angle measure differences of each preparation were totalized (the best possible value here was 0°).

The assessments of the S figure preparations were performed on the top view photographs. The two-dimensional assessment of the S figure included four parameters: overpreparation, underpreparation, accuracy, and the length of inaccurate preparation. The parameter overpreparation measured the total area which was prepared outside the black borderline of the S figure; the parameter underpreparation measured the total area of acrylic which was not removed within the black borderline of the S figure (Fig. 1c). Accuracy was a composite measurement value, combining the overpreparation and underpreparation measurements as follows: 31.656 mm^2^/(31.656 mm^2^ + 0.5 × total area of underpreparation + 2 × total area of overpreparation) [18]. 31.656 mm^2^ was the area within the black borderline of the S figure. Accordingly, the more precise the preparation the closer the accuracy value was to 1. The length of inaccurate preparation measured the total length of the line segments along the black borderline where either overpreparation or underpreparation occurred. The points where the preparation crossed the outer or inner edge of the black borderline served as endpoints for the measurements of overpreparations and underpreparations, respectively.

### Statistical analysis

Statistical analyses were performed with R software (version 3.1.2., R Core Team, R Foundation for Statistical Computing, Vienna, Austria). Spearman rank correlation was used to test the correlation between the results from the conventional visual acuity assessment and the near visual acuity test. Spearman rank correlation coefficients were calculated to assess the intrarater and interrater reliability. To determine interrater reliability in the angle measurements, intra-class correlation coefficients were used. Wilcoxon rank-sum tests were used for the comparison between the preparation measurements from “low visual acuity group” and those from “good visual acuity group.” The relationship between measurements of the preparations carried out with and without magnification loupes was analyzed with Spearman rank correlation coefficients. The level of significance, unadjusted for multiple comparisons, was set at α = 0.05.

## Results

### Visual acuity measurements

The visual acuity of the 59 participants ranged between 0.6 and 1.6. The conventional visual acuity test classified 54 participants in the “good visual acuity group” and 5 in the “low visual acuity group”. The near vision acuity test classified 51 participants in the “good visual acuity group” and 8 in the “low visual acuity group”. The Spearman rank correlation coefficient of the conventional visual acuity test and the near vision acuity test was 0.41, indicating a moderate relationship between the measurements.

### Preparation accuracy assessments

#### Intrarater and interrater reliability

The range of correlation coefficients calculated for the intrarater and interrater reliability was 0.79–1.00, with an average intrarater and interrater correlation coefficient of 0.95 and 0.92, respectively.

#### Impact of magnification loupes on preparation accuracy

Overall, the use of magnification loupes increased the accuracy of the S figure preparations (*p* = 0.0001), and it decreased the total length of the line segments along the black borderline where either overpreparation or underpreparation occurred (*p* = 0.0097).

Participants who were grouped in the “low visual acuity group,” according to the conventional visual acuity test or the near vision acuity test, did not show a statistically significant improvement in either the accuracy or the length of inaccurate preparation when they used magnification loupes for the S figure preparation (*p* ≥ 0.0625). Participants who were grouped in the “high visual acuity group,” according to either test, obtained a higher level of accuracy (*p* ≤ 0.0012) and a shorter length of inaccurate preparation (*p* ≤ 0.0454) when they used magnification loupes for the S figure preparation.

Overall and in both the “good visual acuity group” and the “low visual acuity group,” the use of magnification loupes had no statistically significant effect on the accuracy parameters of the O figure cavity preparations (*p* ≥ 0.1865, *p* ≥ 0.2130, and *p* ≥ 0.0547, respectively).

#### Impact of visual acuity on preparation accuracy

The assessment of the preparations of the S figure showed no significant differences between the “good visual acuity group” and the “low visual acuity group” (*p* ≥ 0.0531), regardless of which visual acuity test was used to classify the participants. The length of inaccurate preparation tended to be greater in the “low visual acuity group,” without reaching statistical significance. Detailed results are given in Table 2.

**Table 2:** Correlation of the visual acuity tests with parameters of preparation precision

Preparation	Parameter	Conventional visual acuity test		Near vision acuity test	
		Spearman rank correlation coefficient	*p* value	Spearman rank correlation coefficient	*p* value
S figure	Accuracy	0.13	0.2562	0.28	0.2976
	Length of inaccurate preparation	− 0.11	0.3173	− 0.22	0.0531
O figure	Depth of the outer cavity	0.17	0.4558	0.18	0.3658
	Evenness of the outer cavity floor	− 0.11	0.0053	− 0.05	0.1401
	Depth of the inner cavity	0.09	0.9417	0.04	0.3080
	Evenness of the inner cavity floor	− 0.06	0.7220	− 0.08	0.8490
	Number of ledges	− 0.20	0.0683	− 0.03	0.0440
	Internal angles of the axial walls	− 0.11	0.5920	− 0.23	0.0937

No significant correlation was observed between the conventional visual acuity test and the accuracy parameters of the O figure cavity preparations apart from the evenness of the outer cavity floor. Participants in the “low visual acuity group” achieved a less even outer cavity floor than those in the “high visual acuity group” (*p* = 0.0053). The near vision acuity test, likewise, showed no correlation with all accuracy parameters of the O figure cavity preparations apart from one: participants grouped in the “low visual acuity group” according to the near visual acuity test left more ledges compared with the “high visual acuity group” (*p* = 0.0440). Table 2 shows these results in detail.

## Discussion

This study showed that untrained undergraduate dental students achieved a higher level of preparation accuracy when they used magnification loupes to complete a two-dimensional preparation task. Magnification had no significant effect, favorable or otherwise, on the accuracy of three-dimensional cavity preparations. Overall, the undergraduates had a high visual acuity, and compared with the conventional visual acuity test, the near vision acuity test proved to be more sensitive in detecting visual deficiencies that are relevant at working distances typical for dentists. Hardly any differences between participants with a visus < 1 and those with a visus ≥ 1 were observed in terms of their preparation accuracy, with only few parameters showing better results in the group with the higher visual acuity.

Based on the findings of the study, the first null hypothesis had to be rejected for the two-dimensional S figure preparation while the data of the three-dimensional O figure preparation did not disprove this null hypothesis. Participants from the “low visual acuity group” achieved a lower level of accuracy in preparing an even outer cavity floor of the O figure and their preparations featured more ledges compared with preparations made by participants from the “good visual acuity group.” Consequently, the second null hypothesis was nullified for these parameters of their preparation precision.

The results from the two-dimensional preparation task corroborate the findings from previous investigations that found that magnification aids may be advantageous for the removal of class I restorations and preparations [19, 20]. The results of the present investigation suggest, however, that magnification offers an advantage for some preparation tasks but not for others.

Some studies report an improvement in procedural quality when magnification is used. A study, performed in a preclinical operative dentistry course, found that using magnification loupes enhanced the acquisition of psychomotor skills required for cavity preparation, which, in turn, lead to faster task completion, less need for assistance, and an improved quality of the students’ performance [11]. Another investigation evaluating the quality of class II cavities, which were prepared by final year undergraduates with and without the aid of magnification loupes, reported that the quality of the cavities prepared with the aid of magnification loupes tended to be rated more favorably than those prepared with unaided vision [10]. However, the proportions of cavities rated as “satisfactory” and “non satisfactory” were not significantly different between the two groups [10].

By contrast, several studies detected no significant impact of magnification on dental preparations. A study assessing the quality of pediatric amalgam preparations found that the use of magnification devices did not raise the quality [15]. Likewise, a study which aimed first and foremost at assessing spontaneous ergonomic changes brought about by the use of magnification loupes reported as incidental finding that magnification loupes had no measurable effect on the quality of class II cavity preparations [13]. The participating students in that study had no previous experience in working with magnification. Furthermore, a study assessed the quality of class I cavity preparations and direct restorations of third-year dental student who carried out the treatment under simulated clinical conditions with and without different magnification devices [16]. It showed that the quality was neither better nor worse when the procedures were carried out with the naked eye [16]. Young prosthodontists, unexperienced in the use of magnification devices, obtained the same level of accuracy in preparations for laminate veneers with and without 2.5× magnification loupes and expert ratings of these preparations were all but identical [14].

It is important to take account of differences in the methodical approaches of these studies. Crucially, the level of (pre-)clinical experience and the degrees of familiarity with magnification vary across studies and these discrepancies likely explain, at least to some degree, the conflicting results regarding the effect of magnification on the procedural quality in restorative dentistry. In the present study, undergraduates at the beginning of their education in operative dentistry were chosen as participants to exclude training effects as confounding factor. This allowed to focus solely on the effect of visual acuity and enhanced vision on procedural quality.

The participants in the present study were at the start of their preclinical training and had no experience in working with magnification loupes apart from the quarter-hour of practice on the duplicate S and O figure. Consequently, their command of the handpiece was not as good as that of skilled users, who can no doubt benefit from magnification equipment for tooth preparation procedures [8, 9]. The level of experience needs to be considered as a limitation of the study because the development of psychomotor skills requires practice. Operators who use magnification devices on a daily basis obtain a significantly higher level of accuracy with magnification compared with the naked eye [20]. However, it takes practice to be proficient in the use of visual enhancement, high-level magnification in particular. Further research is needed to shed light on what kind of specific training in the use of magnification devices results in an optimal learning curve [15].

The results of the present study suggest that magnification loupes increase the accuracy of two-dimensional preparations whereas their effect on the quality of more complex, three-dimensional cavity preparations was scarcely noticeable. The underlying causes for this finding may be that visual control of the diamond bur was always possible in the two-dimensional preparation task while the three-dimensional preparation task arguably relied to a greater degree on tactile sensation and precise eye-hand coordination to control the angulation of the handpiece. Thus, the participants coped with the visual demands in the second task equally well with and without the magnification loupes. Moreover, the fact that hardly any differences were found between participants in the high and low visual acuity group also indicates that excellent vision may not be of the utmost importance for some preparation tasks. This conclusion can, however, only be tentative because the cohort with a low visual acuity was small, which entails a risk of bias. Magnification loupes bring more substantial advantages to accomplish tasks that require total visual control while finer vision contributes less to product improvement of preparations where visual control of the drill tip is restricted.

The present investigation addressed only preparation tasks. However, it is important to consider that the use of magnification may offer more substantial advantages in the steps that follow cavity preparation. For example, magnification makes it easier to detect restoration overhangs, to remove excess resin-based composite, and to trim restorations to the preparation margins [9, 12]. A study, performed under simulated clinical conditions, showed that using 2.3× magnification loupes during the finishing of resin-based composite restorations resulted in a significant reduction of proximal overhangs. There is, moreover, some evidence that suggests the gingival margin quality of proximal fillings is improved when an operating microscope is used during the restoration of the proximal cavities with resin-based composite [21].

Natural visual acuity varies between individuals and it decreases with advancing age [3, 22, 23]. Relevant decreases in visual acuity are frequently observed for the first time around the age of 40 years [17]. This is because people over 40 years are at risk of presbyopia, a normal part of aging [17]. The convenience sample of the present study was biased towards young participants, whose near visual acuity tends to be unimpaired [23]. However, 5/59 students were found to have a low visual acuity, and their preparation skills were almost significantly worse than in the other group. Loupes with 2.5× magnification offer ergonomic rather than optical benefits to younger operators [22]. The low-level magnification of typical Galilean loupes (2.5×) does not significantly improve the visual performance of young operators while, in older people, it can compensate for presbyopic vision deficiencies [22]. Therefore, the results of this study may not be applicable to dentists how rely on magnification loupes to correct their presbyopia.

The level of magnification used in this study was 2.5× because this level, providing a noticeable degree of magnification, is deemed suitable for inexperienced users [19]. A level of magnification around 2.5× is, moreover, commonly used in studies assessing the impact of magnification loupes on restorative procedures [10, 12–14, 19, 24]. However, previous studies demonstrated that Keplerian loupes have a superior visual performance compared with Galilean loupes. Consequently, the results of the present investigation are only applicable to Galilean loupes and not to higher-level magnification.

It is crucial to consider that, in certain clinical situations, magnification may be a detriment to proper treatment [25]. For instance, the use of magnification loupes may increase the risk of iatrogentic enamel damage, in particular to proximal surfaces neighboring mesial class II cavities [24]. Even experienced users of magnification loupes are prone to inadvertently cause such superficial defects, most likely owing to the smaller field of vision when working with magnification loupes [24]. In addition, magnification levels > 2.5× significantly decrease the specificity of visual caries detection [26]. The benefits and disadvantages of magnification should be taught in dental schools and a targeted use of magnification devices would be desirable, from preclinical training to undergraduate patient care and beyond. The question arises whether the routine use of magnification for preparations for direct restorations is advisable. Considering the currently available body of evidence, an answer to this question remains elusive. This gap in our knowledge would benefit from further investigations that explore the merits of magnification in terms of operator ergonomics and procedural quality as well as any drawbacks such as iatrogenic injuries. In any case, it is advisable that dental students and dentists alike regularly undergo professional eye tests.

## Conclusions

The findings of this randomized crossover study, comprising data from dental undergraduates, suggest that loupes with 2.5× magnification increase the accuracy of two-dimensional preparations whereas they have no significant effect, favorable or otherwise, on the accuracy of complex, three-dimensional cavity preparations. Further research, considering postural effects and procedural quality, is needed to determine in which phase and for which tasks magnifications aids should be introduced in undergraduate dental education.

## Data Availability

The dataset used and analyzed during the current study is available from the corresponding author on request.
